# Deep Sequencing of Random Mutant Libraries Reveals the Active Site of the Narrow Specificity CphA Metallo-β-Lactamase is Fragile to Mutations

**DOI:** 10.1038/srep33195

**Published:** 2016-09-12

**Authors:** Zhizeng Sun, Shrenik C. Mehta, Carolyn J. Adamski, Richard A. Gibbs, Timothy Palzkill

**Affiliations:** 1 Department of Pharmacology, Baylor College of Medicine, One Baylor Plaza, Houston, TX 77030, USA; 2 Department of Biochemistry and Molecular Biology, Baylor College of Medicine, One Baylor Plaza, Houston, TX 77030, USA; 3 Human Genome Sequencing Center, Baylor College of Medicine, One Baylor Plaza, Houston, TX 77030, USA

## Abstract

CphA is a Zn^2+^-dependent metallo-β-lactamase that efficiently hydrolyzes only carbapenem antibiotics. To understand the sequence requirements for CphA function, single codon random mutant libraries were constructed for residues in and near the active site and mutants were selected for *E. coli* growth on increasing concentrations of imipenem, a carbapenem antibiotic. At high concentrations of imipenem that select for phenotypically wild-type mutants, the active-site residues exhibit stringent sequence requirements in that nearly all residues in positions that contact zinc, the substrate, or the catalytic water do not tolerate amino acid substitutions. In addition, at high imipenem concentrations a number of residues that do not directly contact zinc or substrate are also essential and do not tolerate substitutions. Biochemical analysis confirmed that amino acid substitutions at essential positions decreased the stability or catalytic activity of the CphA enzyme. Therefore, the CphA active - site is fragile to substitutions, suggesting active-site residues are optimized for imipenem hydrolysis. These results also suggest that resistance to inhibitors targeted to the CphA active site would be slow to develop because of the strong sequence constraints on function.

Metallo-β-lactamases (MBLs) or class B β-lactamases pose a serious threat to β-lactam chemotherapy because of their broad activity spectrum, high rate of horizontal gene transfer among different pathogens, and lack of clinically available inhibitors^1^.

MBLs can be divided into three distinct classes, B1, B2 and B3, based on amino acid sequence homology and the binding of Zn^2+^ at the active site^1^,^2^. A Zn^2+^-binding histidine site is formed by conserved residues His118 and His196 along with His116 (B1 and B3) or Asn116 (B2). The second site (cysteine site) is formed by conserved residues Asp120 and His263 and includes Cys221 (B1 and B2) or His121 (B3). In classes B1 and B3, both Zn^2+^ binding sites must be occupied for maximal activity; however, the B2 class is active only in the mono-zinc form with the Zn^2+^ occupying the cysteine site. Binding of Zn^2+^ to both sites inhibits the activity of B2 enzymes.

CphA β-lactamase, identified from the Gram-negative opportunistic pathogen *Aeromonas hydrophila*^3^, is a subclass B2 MBL and is non-competitively inhibited by the binding of the second Zn^2+^ with a *K*_i_ value of 46 μM^4^. Unlike the broad-spectrum of antibiotics hydrolyzed by B1 and B3 MBLs, CphA efficiently hydrolyzes only carbapenem antibiotics such as biapenem, imipenem, and meropenem^5^,^6^.

Crystal structures of both the apo form of CphA and its complex with a hydrolysis intermediate of biapenem have been determined^7^. Both structures contain a single zinc ion that is located in the cysteine site. A comparison of the structure of CphA to other MBL structures revealed that the active site of CphA is narrower and deeper than that of other MBLs, and appears optimized for carbapenem binding and hydrolysis^7^. Based on the structure of the enzyme-intermediate complex and theoretical investigations with a quantum mechanical/molecular mechanical method and density functional theory, a reaction mechanism for CphA has been proposed^7^,^8^,^9^,^10^ (Fig. 1A). A water molecule in the active site is held through interaction with His118 and Asp120. One of these two residues serves as a general base to activate the water molecule for attack on the carbonyl carbon of the β-lactam ring. Meanwhile, the carbonyl group is polarized by interaction of the carbonyl oxygen with His196. After cleavage of the C–N bond, the anionic nitrogen is stabilized by an interaction with the Zn^2+^ and protonated by a water molecule bound to His118 and Asp120 or Zn^2+^.

The amino acid sequence requirements for the function of CphA and other MBLs have been studied by site-directed mutagenesis^11^,^12^,^13^,^14^,^15^. However, comprehensive, saturation mutagenesis of active site positions has been limited to the subclass B1 IMP-1 enzyme^16^. For these experiments, individual codons for active site residues in IMP-1 were randomized to create libraries containing all possible amino acid substitutions. Each library was then subjected to a genetic selection for those variants that allowed growth of *E. coli* in the presence of various β-lactam antibiotics. DNA sequencing of the functional mutants revealed the importance of each residue position for catalysis and substrate specificity^16^. The power and resolution of this approach has recently been greatly expanded by next generation sequencing technologies, which allow the rapid determination of the sequence of thousands of functional mutants simultaneously^17^. We have recently used this randomization, functional selection and deep sequencing approach to determine the amino acid sequence requirements for ampicillin resistance for each residue in the TEM-1 β-lactamase^18^.

This study expands on the codon randomization, functional selection, and deep sequencing approach by constructing single codon randomized libraries for residue positions in and near the active site of CphA (Fig. 1B) and selecting for growth of *E. coli* in the presence of increasing concentrations of imipenem, a carbapenem antibiotic. Sequence information for the functional mutants was obtained by deep sequencing. The frequency of occurrence of each amino acid at each randomized position identified multiple positions at which the wild-type amino acid residues predominate among the functional clones. Functional and biochemical analyses of specific mutants confirmed their importance in the maintenance of imipenem-hydrolyzing activity or protein stability of CphA. The results indicate that there are stringent sequence requirements in the active site of CphA in that residues in direct contact with zinc or substrate as well as many second shell residues that do not contact substrate do not tolerate amino acid substitutions. Therefore, the active-site residues in CphA appear optimized for imipenem hydrolysis. This study also suggests that resistance to inhibitors targeted to the CphA active site would be slow to develop because of the strong sequence constraints on function.

## Results

### Deep sequencing of randomized single codon libraries of residue positions in and near active site of CphA

**Figure 1:**
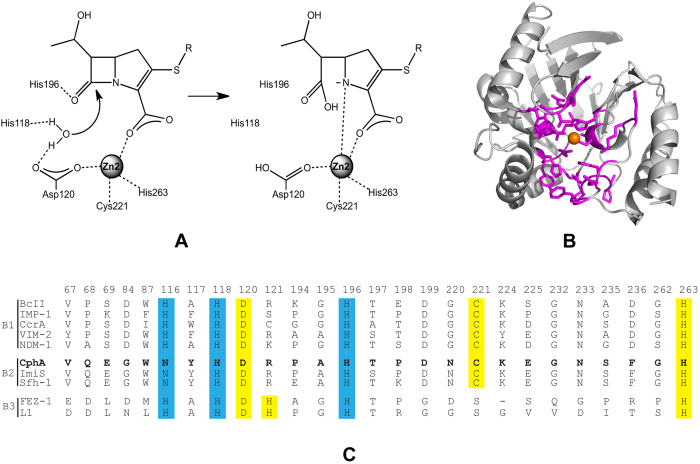
Active site residues of metallo-β-lactamases. (**A**) Schematic representation of carbapenem substrate binding and anionic intermediate stabilization in the active site of mono-zinc metallo-β-lactamase CphA. An active site water interacts with Asp120 and His118 and is activated for attack on the carbonyl carbon of the carbapenem, whose carbonyl group is polarized by interaction of the carbonyl oxygen with His196. After C-N bond cleavage, anionic nitrogen is stabilized by interactions with the zinc ion. (**B**) Diagram of the CphA β-lactamase structure highlighting active site residues for which random mutant libraries were created. The zinc atom is represented as an orange sphere. The figure was rendered with the Pymol program using coordinates from the Protein Data Bank accession code 1X8G ^7^. (**C**) Sequence alignment of representative metallo-β-lactamases from subclasses B1, B2, and B3. The residues in blue boxes and yellow boxes indicate the histidine and cysteine zinc binding site residues, respectively. CphA residues are in bold.

Randomized, single codon mutation libraries were constructed targeting 26 residues in and near the active site based on the structure of mono-zinc form of CphA^7^ (Fig. 1B). Functional mutants were obtained by introducing each of the libraries into *E. coli* and selecting for growth of colonies in the presence of multiple concentrations of the carbapenem antibiotic, imipenem (0.1, 0.2, 0.4, and 0.8 μg/ml). This was designed to allow the selection to distinguish sequence requirements for minimal as well as wild-type levels of CphA function. Functional clones from each library for each imipenem selection were pooled and plasmid DNA was prepared from each pool. A total of 130 pools (26 naïve library pools and 104 imipenem-selected pools) were individually amplified with forward primers containing unique barcode sequences and non-barcoded reverse primers. The tagged amplicons were gel-purified, gathered into one final pool with equal amounts of each PCR amplicon and sequenced in one batch using Illumina MiSeq sequencing.

### Analysis of deep sequencing data

Quality analysis of the sequencing data showed that the per base sequence quality score was higher than 30 except for positions at the extreme end of the reads (after 140 bp) (Fig. S1). This quality score indicates the probability of error is lower than 1 in 1000 at the base position. The PCR amplicons for sequencing were designed to place the randomized codons for the libraries 30–40 bp from the 5′ terminus and therefore the randomized codons were always in a region of high quality sequence. The sequencing data was analyzed with a custom Perl script which binned reads to the appropriate libraries and imipenem selections by recognizing both the barcode sequence and the sequences flanking the randomized codon (Methods). A total of 1.8 × 10^7^ reads could be assigned for the 130 experiments (140,000 reads for each experiment on average). A custom Python script was then used to count the occurrence of each amino acid type in each library for each selection, as shown in Table S1.

For most libraries, each amino acid type was observed at a comparable frequency in the naïve libraries. However, for residue positions Gly84 and Gly262, the wild-type residue glycine showed 2-fold and 5-fold higher occurrence frequency than any other amino acid type and represented 28% and 39% of the total read number in the naïve library (Table S1). This bias for glycine codons might be introduced during PCR-amplification of the pooled library DNA since the bias was not found at either position in Sanger sequencing results of single colonies from naïve library plates (data not shown).

After selection with 0.1 μg/ml imipenem, the fraction of sequences containing glycine increased to 52% of the total read number at residue position Gly262, implying that glycine is preferred at the position even when only low level carbapenemase function is required. When the imipenem selection concentration was increased to 0.2 μg/ml, wild-type residues became predominant in libraries for the Asp120 and His263 residues in the mono-zinc binding site as well as His118 and His196, which have been implicated in the catalytic mechanism of the mono-zinc enzyme^7^,^12^,^19^. In addition, the wild-type residue became predominant at other positions involved in substrate binding (Lys224) or conserved across MBL enzymes (Gly232) (Fig. 1C and Table S1). In these experiments, wild-type amino acids occur at a frequency at least 10-fold higher than that of any other amino acid type at the same position (Table S1). For the other zinc-binding residue Cys221, the wild-type amino acid occurred dramatically more frequently than any other amino acid only after the imipenem selection concentration reached 0.4 μg/ml (Table S1), which was also the case for residues Gly84, Asn116, Thr197, Asp199, and Asn233 (Table S1). When the imipenem selection concentration was further increased to 0.8 μg/ml, wild-type amino acids at Glu69, Tyr117, Arg121, and Asn220 also became prominent (Table S1). However, for nine positions, including Val67, Gln68, Trp87, Pro194, Ala195, Pro198, Glu225, Ser235, and Phe236, even the highest imipenem concentration (0.8 μg/ml) did not select for predominately the wild-type amino acids (Table S1).

Taken together, the results indicate that at the highest imipenem concentration, which selects for phenotypically wild-type levels of CphA function, the wild-type residue predominates at 17 out of 26 positions randomized, i.e., the wild-type residue cannot be substituted at the majority of positions without some loss of function.

### Sequence logos of naïve and imipenem-selected libraries

**Figure 2:**
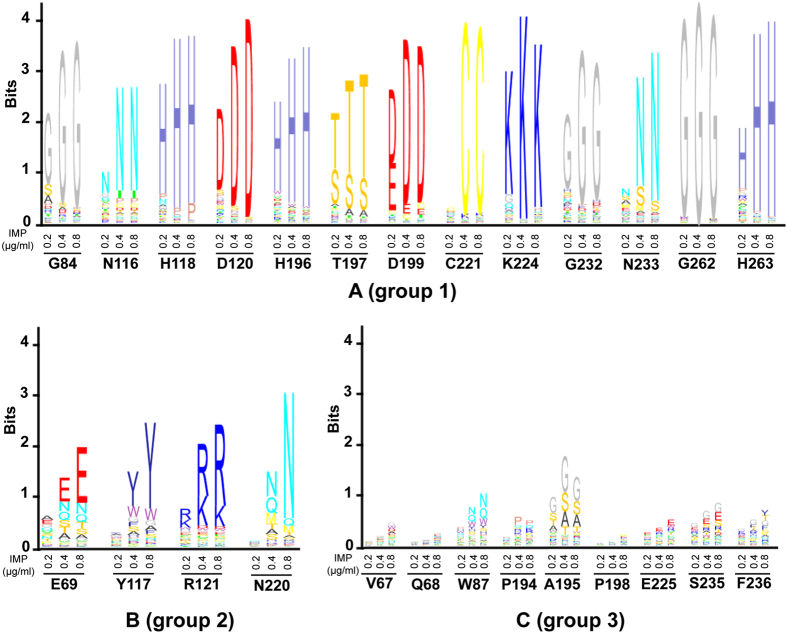
Sequence logos for group 1 (**A**), group 2 (**B**) and group 3 (**C**) residue positions. (**A**) For group 1 positions, the wild-type residue dominates among sequences from populations selected at both high and low imipenem concentrations. (**B**) For group 2 positions, wild type sequences predominate in selections at high imipenem concentrations but other amino acid types are found at low imipenem concentrations. (**C**) For group 3 positions, the wild-type residue does not predominate even in selections at high impenem concentrations.

In order to visualize sequence conservation among mutants from the imipenem-selected libraries, sequence logos were created based on the deep sequencing results from imipenem-selected libraries. Since 0.1 μg/ml imipenem did not result in any discernible selection of specific amino acids in libraries for all residue positions except Gly262 (Table S1), sequence logos for 0.1 μg/ml imipenem selected libraries were omitted. Sequence logos for libraries selected by 0.2, 0.4, and 0.8 μg/ml imipenem are shown in Fig. 2.

Three groups of residues can be distinguished based on the sequence logos in Fig. 2. In the first and largest group (Fig. 2A), the wild-type residue predominates among the functional sequences even at low concentrations (0.2–0.4 μg/ml) of imipenem used for selection. The dominance of the wild-type sequence at lower imipenem concentrations indicates that there is a large decrease in function relative to wild type for substitutions at these positions, i.e., substitutions at these positions result in modest or no CphA function. This group includes the zinc binding residues in the cysteine site as well other residues that are implicated in substrate binding and catalysis.

The second group (Fig. 2B) includes positions where residues in addition to wild type are observed among functional sequences selected at low imipenem concentrations. At the highest imipenem concentration, however, the wild-type residue predominates. For these positions, the wild-type residue provides the highest levels of function but other substitutions are partially functional. This group consists of the second shell positions that do not directly contact substrate or zinc and includes Glu69, Tyr117, Arg121, and Asn220.

The third group (Fig. 2C) consists of positions where no residue type predominates even at the highest imipenem concentration used for selection. For these positions, sequence requirements for function are not stringently specified and several residue types function as well as wild type. For these positions, it can be concluded that the precise chemical characteristics of the side chain are not required for CphA function. It should be noted that although several different residue types provide for function at group 3 residues, not all substitutions are allowed (Table S1). For example, cysteine substitutions are rare at many of these positions (Table S1). Group 3 positions are largely found distant from the bound zinc and catalytic residues. However, Val67 and Trp87 form a hydrophobic wall of the active site and are in position to interact with bound substrate. Nevertheless, the sequencing results suggest that several different residue types can perform this function at positions 67 and 87.

### Impact of amino acid substitutions on CphA function

The deep sequencing results of the CphA randomized libraries indicated 17 residue positions in and near the active site that could not be substituted when evaluated at high imipenem concentrations. To validate the results using a different method, site-directed mutagenesis was performed to measure the effects of amino acid substitutions at these positions on CphA function in conferring imipenem resistance. The wild-type amino acid residue at these positions was substituted by the second most frequently occurring amino acid and/or those with similar physicochemical properties as the wild-type residue. In addition, for those residues that are conserved only in subclass B2 enzymes such as Glu69, Gly84, and Asn220, the wild-type residues were also substituted by amino acids found in the conserved counterpart in B1 enzymes.

The imipenem resistance levels (minimum inhibitory concentrations, MIC) of *E. coli* cells transformed with empty vector and the wild-type CphA expression vector were determined to be 0.25 μg/ml and 2 μg/ml, respectively. As expected, substitution of the Zn^2+^-binding residue His263 with alanine decreased imipenem resistance to a level similar to that of the no β-lactamase control (0.38 *vs* 0.25 μg/ml) (Table 1). Any amino acid substitutions at important, non-chelating residues also decreased CphA function in proportion to their frequency of occurrence in the deep sequencing experiment. For example, amino acid substitutions at residue positions Thr197 and Asn233 from group 1 (Fig. 2A) and Tyr117 and Arg121 from group 2 (Fig. 2B) by residues that displayed the highest occurrence frequency besides the wild-type residue (e.g. T197S, N233S, Y117W, and R121K) led to a 2- to 3-fold decrease in the imipenem MIC of *E. coli* containing the mutant CphA enzyme (Table 1). However, substitutions at the same positions by residues showing a low frequency of occurrence resulted in dramatically reduced resistance (e.g. T197L, N233Q, and Y117V) or a lack (e.g. R121A) of imipenem resistance (Table 1).

The deep sequencing results indicate 9 residue positions in group 3 that allow for amino acid substitutions with a frequency of occurrence similar to wild-type CphA even for the selection at high imipenem concentrations (0.8 μg/ml) (Fig. 2C). In order to confirm these findings, residues at 3 of these positions (Ala195, Pro198, and Ser235) were substituted by amino acids that displayed a similar frequency of occurrence as wild type in the 0.8 μg/ml imipenem selection experiments. MIC determinations showed that none of these amino acid substitutions significantly compromised imipenem resistance function of CphA (Table 1).

In summary, the critical roles of residue positions identified as important by deep sequencing of imipenem-selected libraries were confirmed by MIC determinations. Any amino acid substitution at these positions decreased imipenem resistance levels provided by CphA. In addition, amino acid substitutions at positions identified as freely substituted in deep sequencing experiments retained wild-type resistance levels.

### Impact of amino acid substitutions on imipenem-hydrolyzing activity and cellular expression of CphA

The antibiotic resistance level provided by wild-type CphA and mutants is a function of the enzyme’s ability to hydrolyze the drug as reflected in its kinetic parameters (*k*_cat_, *k*_cat_/*K*_m_) for imipenem hydrolysis as well as the effects of the mutation on the steady-state level of the protein in the periplasm. The *in vitro* kinetic parameters *k*_cat_ and *k*_cat_/*K*_m_ were determined for purified wild-type and several mutant CphA enzymes and the steady-state expression levels were measured by western blot using an antibody against a C-terminal StrepII tag that was present in the wild-type and mutant enzyme libraries.

**Figure 3:**
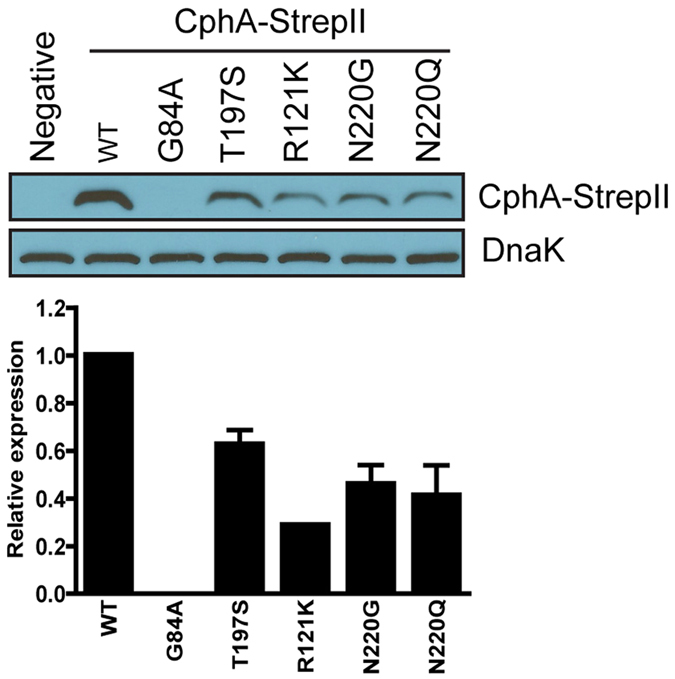
*In vivo* steady-state protein levels of wild-type and CphA mutants. Steady-state protein levels of StrepII-tagged wild-type and CphA mutants in recombinant *E. coli* were determined by SDS-PAGE of whole cell lysates followed by immunoblotting with an anti-StrepII tag monoclonal antibody conjugated to horseradish peroxidase (HRP). Constitutively expressed DnaK (~70 kDa) was used as a loading control and probed with anti-DnaK monoclonal antibody and HRP-conjugated secondary antibody. The hybridization signal for wild-type and mutant CphA-StrepII and DnaK was quantified by densitometry. The signal for CphA-StrepII was normalized to that for DnaK in the same sample. Protein levels of mutant CphA-StrepII are expressed in the bar graph relative to that of the wild-type protein, which was set as 1. Quantification data are based on three independent experiments and a representative blot is shown.

Enzyme kinetic analyses of imipenem hydrolysis by mutants at group 1 residue positions that cannot be substituted even at low concentrations of imipenem as indicated by deep sequencing (Fig. 2A) generally showed that these enzymes are much less effective catalysts than wild-type CphA (Table 1). For example, the G84D, T197L, K224R, G232A, N233Q and G262A mutants all exhibit ~10-fold reduced catalytic efficiency (*k*_cat_/*K*_m_) for imipenem hydrolysis while an alanine substitution at the zinc-binding residue His263 results in a >100-fold decrease (Table 1). However, the G84A and T197S substitutions are exceptions in that they result in a less than 2-fold decrease in *k*_cat_/*K*_m_ for imipenem hydrolysis, which is unexpected considering the sequencing results. As seen in Fig. 3, however, evaluation of steady-state protein expression levels in *E. coli* by immunoblotting showed that G84A and T197S are expressed at reduced levels, presumably due to decreased stability and proteolysis, which provides an explanation for the low numbers of substitutions in the deep sequencing results.

Enzyme kinetic analysis of mutants with substitutions at group 2 positions that exhibit stringent sequence requirements at high imipenem concentrations indicate that all exhibit reduced *k*_cat_/*K*_m_ values for imipenem hydrolysis except for R121K, N220G and N220Q (Table 1). Analysis of steady-state protein expression levels reveals reduced expression of the mutants compared to wild type (Fig. 3), which could contribute to the lower *in vivo* activity of these mutants. Finally, among those group 3 positions that deep sequencing results indicated could be substituted even at high imipenem concentrations (Fig. 2C), the P198E mutant enzyme was tested and exhibited a *k*_cat_/*K*_m_ value similar to wild type CphA, as expected.

In summary, the residue positions in CphA identified as important by the deep sequencing data are involved in imipenem catalysis or enzyme stability. Disruption of either role decreases the imipenem resistance function of CphA.

## Discussion

**Figure 4:**
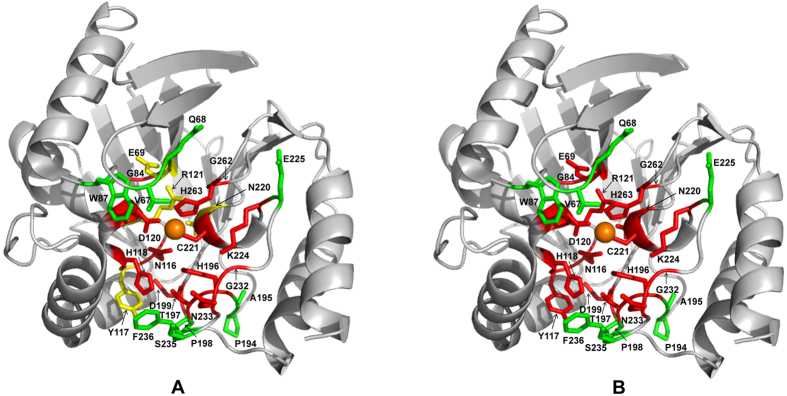
Diagram of the CphA β-lactamase structure showing the location of residue positions randomized in this study. (**A**) Group 1, 2, and 3 residues are labeled red, yellow, and green, respectively, while the zinc atom is represented as an orange sphere. (**B**) Active site residues are colored according to whether positions can tolerate substitutions and retain wild-type levels of CphA function. For residues shown in red, amino acid substitutions reduce enzyme activity relative to wild type. Residues shown in green can be substituted and retain wild type levels of function. The figure was rendered with the Pymol program using coordinates from the Protein Data Bank accession code 1X8G ^7^.

Deep sequencing of functionally selected random mutant libraries has been recently utilized for exploring the structure, function and evolution of various proteins^17^,^18^,^20^,^21^,^22^,^23^,^24^. In this study, 26 residue positions in or near the active site of *Aeromonas hydrophila* CphA metallo-β-lactamase were subjected to randomization, functional selection, and deep-sequencing to systematically evaluate the amino acid sequence requirements for CphA function. Deep sequencing revealed the number and type of each of the surviving alleles in the antibiotic-resistant population after selection on different concentrations of imipenem. Residue positions could be placed in three groups based on the sequencing data. Group 1 consisted of positions with strict sequence requirements for the wild-type residue even at low concentrations of imipenem (Fig. 2A). Group 2 consisted of positions with strict sequence requirements for the wild-type residue at high imipenem concentrations but with substitutions with partial function present in the populations selected at low imipenem concentrations (Fig. 2B). Group 3 consisted of positions where multiple residue types were present in the populations selected at high concentrations of imipenem (Fig. 2C). The residues in each of these groups are shown colored red (group 1), yellow (group 2), and green (group 3) on the CphA structure in Fig. 4A.

Residue positions in the group 1 with stringent sequence requirements include the Zn^2+^-binding ligands (Asp120, Cys221, and His263), as well as Asn116, His118, His196, and Lys224, whose functions have been well-defined^7^,^8^,^9^,^11^,^12^,^15^,^19^, and other residues (Gly84, Thr197, Asp199, Gly232, Asn233, and Gly262) whose functions were previously unexplored in CphA. Consistent with their involvement in binding the catalytic Zn^2+^, any amino acid substitutions at the Asp120, Cys221, or His263 residue positions strongly decreased the enzyme activity and resistance function of CphA against imipenem, which is too low to support cell growth in the presence of moderate or even low levels of imipenem (Fig. 2A and Table S1). Residue His118 has been suggested to bind and also possibly activate the hydrolytic water molecule in the active site^7^ while His196 and Lys224 are directly involved in substrate binding by forming a hydrogen bond with the carbonyl oxygen of the β-lactam and electrostatically interacting with C-3 carboxylate group of carbapenems, respectively^7^. Therefore, any amino acid substitution at these residue positions decreases CphA activity and function, which is consistent with site-directed mutagenesis studies^12^. Interestingly, a K224R substitution, in which the positive charge is retained, also resulted in a large decrease in imipenem catalytic efficiency of CphA (Table 1), as has been observed for subclass B1 MBLs CcrA and IMP-1^25^,^26^. This suggests that the K224R substitution may have an altered position of the positively charged side chain and a weaker interaction with the carboxylate group, which is supported by a several fold higher *K*_*m*_ value of the mutant compared to wild-type CphA (Table 1).

**Figure 5:**
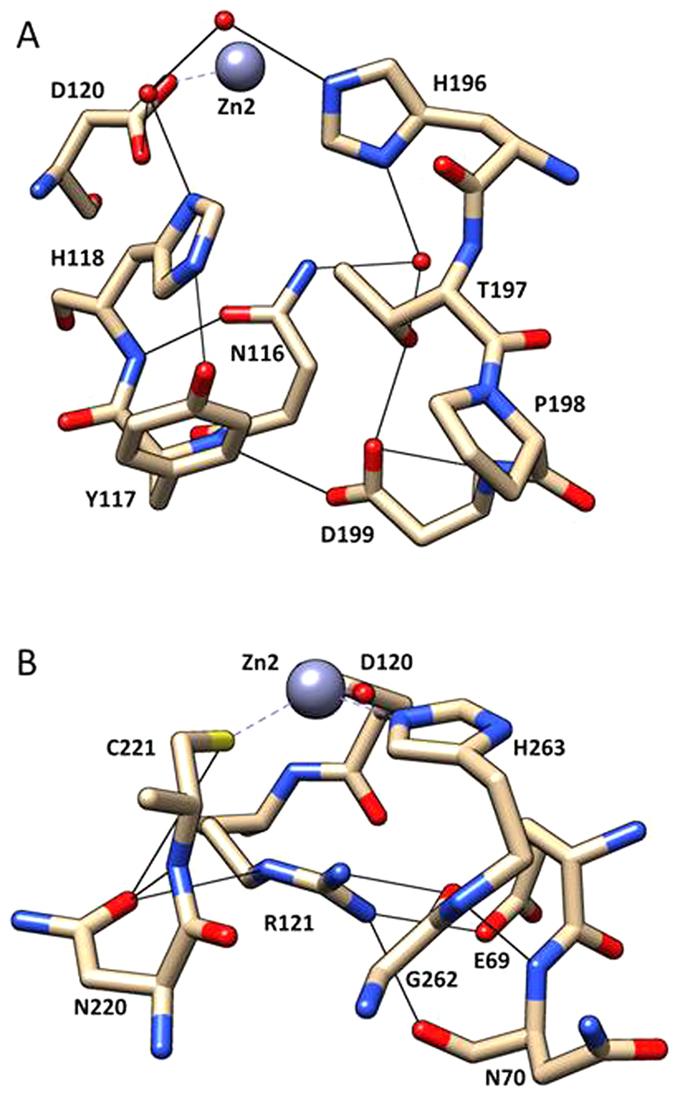
Hydrogen bond networks near His118 and Arg121. Carbon is shown in tan, nitrogen is blue and oxygen is red. Water molecules are shown as red spheres and zinc is indicated as a gray sphere. Hydrogen bonds are depicted as black lines. (**A**) Schematic illustration of the hydrogen bond network near residue His118. Asn116, His118, Asp120, His196, Thr197 and Asp199 are group 1 residues that cannot be substituted based on deep sequencing results. Tyr117 is a group 2 residue that cannot be substituted during selections at high imipenem concentrations and Pro198 is a group 3 residue that tolerates substitutions while retaining function. (**B**) Schematic illustration of the hydrogen bond network near residue Arg121. Asp120, Cys221, Gly262 and His263 are group 1 residues that cannot be substituted based on deep sequencing results. Glu69, Arg121 and Asn220 are group 2 residues that cannot be substituted during selections at high imipenem concentrations. Asn70 was not mutagenized in this study.

In contrast to the highly important residues discussed above, the group 1 residues Asn116, Thr197, and Asp199 do not directly bind substrate, catalytic Zn^2+^, or the catalytic water molecule. However, the structure of CphA in complex with a hydrolyzed carbapenem indicated that their side chains are involved in the formation of a hydrogen bonding network (Fig. 5A) that helps shape the active site in an arrangement that facilitates the formation of hydrogen bond between imidazole side chain of His196 and carbonyl oxygen of the β-lactam ring^7^. Although residues Asn116 and Asp199 are not tolerant of amino acid substitutions^11^ (Fig. 2A), an isosteric serine substitution at position Thr197 modestly decreased the imipenem hydrolysis activity of CphA (Table 1). However, substitution of Thr197 with a hydrophobic residue (Leu) dramatically decreases the catalytic efficiency of CphA (Table 1). Another residue that contributes to this hydrogen bond network is Tyr117 (Fig. 5A). This residue belongs to group 2 in that substitutions are found among the mutant populations selected at low but not high impenem concentrations indicating the wild-type tyrosine is required for high-level imipenem hydrolysis. The hydroxyl group of the Tyr117 side chain forms a hydrogen bond with His118, which may position the histidine side chain for activation of the catalytic water. In addition, Tyr117 forms an aromatic interaction with His118 (Fig. 5A). The importance of an aromatic interaction is supported by the finding that substitution of Tyr117 with valine is more deleterious than phenylalanine or tryptophan substitutions with regard to catalytic efficiency (Table 1).

There are three glycine residues (Gly84, Gly232, and Gly262) in and near the active site of CphA and their functional roles have not previously been explored. Each of these residues belongs to group 1 and cannot be substituted even at low imipenem concentrations indicating an important role in CphA function. These residues likely play a steric role in that, based on the structure, substitutions would be expected to clash with other residues (Gly84) or interfere with either substrate (Gly232) or zinc (Gly262) binding. Consistent with these roles, a G84A substitution exhibits low protein expression levels (Fig. 3) while enzymes with G232A or G262A substitutions exhibit lower catalytic activity than wild-type CphA (Table 1). Gly232, together with Asn233, are implicated in substrate binding by forming a loop at the entrance of the CphA active site^7^. Asn233 is also a group 1 residue that, based on sequencing data, cannot be substituted (Fig. 2A). Upon substrate binding, a conformational change occurs in the loop, which results in an enhanced interaction with substrate and closure of the entrance to the active site^7^. Substitutions at either Gly232 or Asn233 increased *K*_*m*_ by at least two-fold (Table 1), supporting the role of these residues in substrate binding. Gly262 is located next to the Zn^2+^-chelating residue His263 and its flexibility allows the protein backbone to turn by 90° so that His263 adopts a conformation that facilitates Zn^2+^ coordination^7^. A G262A substitution decreased CphA catalytic efficiency by more than 15-fold (Table 1), possibly due to interfering with zinc binding to His263 and disruption of a hydrogen bond formed with side chain of Arg121.

Group 2 positions include residues Glu69, Arg121 and Asn220 that are second-shell residues that participate in a hydrogen-bond network situated below the bound zinc in the cysteine site (Fig. 5B). This network also includes the aforementioned group 1 residue Gly262 as well as the main chain carbonyl oxygen of Asn70 (which was not a target of mutagenesis). Glu69, Arg121 and Asn220 tolerate substitutions at low but not high imipenem concentrations (Fig. 2B). The guanidinium group of Arg121 residue is situated directly below the bound zinc. The guanidinium group forms a salt bridge with Glu69 and also forms hydrogen bonds with the main chain carbonyl oxygens of Asn70 and Gly262 as well as the side chain of Asn220 (Fig. 5B)^7^. These interactions link together residues from different sections of the active site. Substitution of Arg121 by Lys reduced CphA catalytic efficiency by 2- to 3-fold; whereas an Ala substitution had a more drastic effect, decreasing *k*_*cat*_/*K*_*m*_ by 50-fold (Table 1). Clearly a positively charged residue is important at position 121. The effects of substitutions at Glu69 are more complex. Although Glu69 forms a salt bridge with Arg121, an Asp substitution is more deleterious than a Ser substitution for enzyme activity (Table 1). Interestingly, serine is the most common residue at this position in subclass B1 enzymes (Fig. 1C). The E69S mutation may create new interactions with a nearby water molecule or residues such as Thr86 to maintain a catalytically active conformation of CphA, although this requires structural confirmation.

Residue positions Val67, Gln68, Trp87, Pro194, Ala195, Pro198, Glu225, Ser235, and Phe236 are group 3 residues and are tolerant of multiple amino acid substitutions even when high concentrations of imipenem were used for selection (Fig. 2C and Table 1). Among them, the hydrophobic side-chains of Val67 and Trp87 are involved in the formation of a ‘hydrophobic wall’ that defines one side of the active site of the CphA enzyme^7^. However, some amino acid residues with polar side chains (e.g., V67S, W87N, and W87Q) were as functional as wild-type at these positions (Table S1). Phe236 also forms a hydrophobic wall of the active site near His118 and this residue can also be substituted. Therefore, these residue positions that tolerate substitutions such as Val67, Trp87, and Phe236 seem to play a structural role that narrows the active site but does not require precisely specified side chain characteristics to perform the function. As is evident in Fig. 4A, other group 3 residues are located on the periphery of the active site and do not make direct interactions with substrate or participate in hydrogen bond networks that shape the active site structure. Group 3 residues that tolerate substitutions may represent targets for evolution of variants that exhibit altered activity or substrate specificity as mutations at these positions would not be expected to alter the core hydrolysis function of the enzyme.

In summary, deep sequencing of random mutant libraries selected for imipenem resistance together with biochemical analyses allowed an assessment of the sequence requirements for CphA β-lactamase function. The residues can be placed in three groups based on the prevalence of substitutions in the sequenced populations of random mutants at various imipenem concentrations as described above (Fig. 2). When considering the sequence requirements for wild-type levels of CphA activity, however, the residues fall into two groups with 17 out of the 26 positions examined exhibiting stringent sequence requirements where only the wild-type residue provides high-level function (Fig. 4B). With the exception of two positions (Val67 and Trp87), these residues encompass all of the residues that could make direct contact with zinc or substrate as well as second shell residues that form hydrogen bonding networks that shape the active site and position the residues in direct contact with zinc or substrate. Therefore, the sequence of active-site residues appears optimized for carbapenem hydrolysis and fragile to amino acid substitutions. These findings suggest that resistance to inhibitors targeted to the CphA active site would be slow to develop because of the strong sequence constraints on function.

A comparison of the sequence requirements for CphA with those inferred from previous codon randomization and functional selection studies of active site residues in the IMP-1 metallo-β-lactamase suggest more information is required for carbapenem hydrolysis by CphA in that it exhibits more stringent sequence requirements^16^. IMP-1 is a subclass B1 enzyme that contains 2 zinc atoms in the active site and a broad substrate profile that includes penicillins, cephalosporins and carbapenems^1^. In the IMP-1 study, 29 residue positions in and around the active were randomized and functional mutants were selected with representative penicillins, cephalosporins and carbapenems. Sequencing of functional mutants indicated that approximately 25% of the residues (7/29) required the wild type residue for hydrolysis of all β-lactams tested^16^ (Table S2). In comparison, approximately 65% (17/26) of the active site residues in CphA require the wild type residue for high level carbapenem hydrolysis. With regard to enzymes other than metallo-β-lactamases, codon randomization and functional selection studies have also been performed on active site residues of the class A TEM-1 β-lactamase^18^,^24^ and the class C P99 β-lactamase^27^. The TEM-1 and P99 β-lactamases utilize an active site serine as a nucleophile to attack the carbonyl carbon of the amide bond in the β-lactam ring and proceeds through a covalent acyl-enzyme intermediate that is subsequently hydrolyzed by a base-activated water molecule^28^. Codon randomization followed by a selection for ampicillin resistance was used to assess the tolerance of TEM-1 residue positions to substitutions^18^,^24^. The entire TEM-1 coding sequence has been randomized, however, evaluation of 31 positions in and near the active site reveals that approximately 30% (9/31) do not tolerate substitutions (Table S2). Codon randomization followed by a selection for cephalosporin hydrolysis revealed that approximately 40% (9/21) active site positions tested in P99 did not tolerate substitutions^27^(Table S2). Therefore, the active site of this enzyme is less tolerant of substitutions than IMP-1 and TEM-1 but more tolerant than CphA β-lactamase.

ODCase catalyzes the decarboxylation of orotidine 5′-monophosphate (OMP) to uridine 5′-monophosphate (UMP) during the biosynthesis of pyrimidine nucleotides. ODCase has been extensively studied owing to its high catalytic proficiency, which is defined as *k*_cat_/*K*_m_ divided by the rate of the spontaneous reaction in neutral solution (*k*_non_)^29^. ODCase is one of the most proficient enzymes known with a (*k*_cat_/*K*_m_)/*k*_non_ value on the order of ~10^23^ M^−1^. Codon randomization and selection experiments on 24 residues in and near the active site of ODCase revealed that 25% (6/24) of the positions require the wild type amino acid for function under stringent selection conditions^30^ (Table S2). Therefore, the IMP-1 β-lactamase and ODCase exhibit similar sequence requirements in terms of tolerance to substitutions, P99 β-lactamase is less tolerant than IMP-1 and ODCase, while the CphA enzyme appears the most fragile to substitutions. Future studies of other enzymes will provide insight into the generality of these observations.

The CphA β-lactamase hydrolyzes carbapenems but not other classes of β-lactams. It is possible that mutants within the 26 CphA random libraries could alter the substrate specificity of the enzyme. This possibility was preliminarily tested by evaluating whether the E69S and G84D substitutions alter the substrate specificity of CphA. These residues are conserved within each subclass of MBLs but are different residues in each subclass (Fig. 1). Thus, positions 69 and 84 are Glu and Gly, respectively, in subclass B2 enzymes but are Ser and Asp, respectively, in subclass B1 enzymes. Ampicillin and cefotaxime were used as the representative penicillin and cephalosporin substrates. It was found that E69S mutant did not exhibit any detectable activity against either β-lactam. However, although G84D mutant did not show any ampicillin hydrolysis activity, it did show a significant hydrolysis activity against cefotaxime with *k*_cat_/*K*_*m*_ = 0.0003 ± 0.00002 sec^−1^ μM^−1^. Therefore, substitutions in CphA can alter the substrate specificity. Experiments are in progress to systematically examine the CphA random libraries for mutations that alter substrate specificity.

## Methods

### Bacterial strains and vectors

*E. coli* XL1-Blue (Stratagene) and *E. coli* BL21 (DE3) were used as the host strains for the construction of codon randomization libraries and for over-production of wild-type or mutant CphA enzyme, respectively. The plasmid pTP470, which encodes chloramphenicol resistance and is a *lacI*^*q*^-deletion derivative of pTP123 described previously^31^, contains the gene for CphA, whose expression is under the control of IPTG-inducible *trc* promoter. The Strep-tagII sequence is included at the C-terminus of CphA to allow for monitoring the expression of CphA in *E. coli* by immunoblotting with anti-Strep-tagII antibody^14^. For over-production of CphA for subsequent purification, the CphA-pET29a plasmid was constructed by insertion of a PCR product encoding wild-type CphA into the *NdeI*/*XhoI* restriction sites of pET29a (Novogen). Site-directed mutagenesis using the Quick-Change method was performed on CphA-pET29a and CphA-StrepII-pTP470 to obtain expression vectors for CphA(-StrepII) mutants^32^.

### CphA codon randomization library construction

CphA single codon random libraries were constructed using the method modified from that employed previously by Huang *et al*.^33^ and Sullivan *et al*.^34^. Briefly, to eliminate any wild-type CphA contamination, a *Xho*I restriction site was first inserted near the target codon for randomization. The insertion of the *Xho*I recognition sequence was designed to also introduce a frameshift mutation in the CphA gene to ensure the insert mutant is non-functional. The *Xho*I insert mutant was then used as the template for randomization of each codon. The codon for the target residue was substituted by NNS (where N is any nucleotide type and S is G or C) so that codons for all 20 amino acids were represented in the library. The resulting mutagenesis reactions were treated with *Dpn*I and *Xho*I to eliminate non-mutagenized plasmids and transformed into *E. coli* XL1-Blue by electroporation. A minimum of 300 colonies were pooled for each library construction and used for preparation of plasmid DNA to obtain a single codon randomization library.

### Selection of CphA functional mutants on agar plates containing imipenem

In order to select for functional mutants, 100 ng of plasmid library DNA was transformed into *E. coli* XL1-Blue by electroporation and the bacterial cells were spread on LB agar plates containing 0.5 mM IPTG and 0.1, 0.2, 0.4, and 0.8 μg/ml imipenem. The transformation cultures were diluted appropriately to produce approximately 1,000 colonies on each selection plate. The resulting colonies were pooled and used for plasmid preparation.

### Preparation of CphA library samples for Illumina deep sequencing

The plasmid DNA obtained from pooled colonies after selection for imipenem resistance as well as the plasmid DNA from the naïve, unselected starting library, was used as template DNA for PCR reactions in preparation for sequencing. PCR primers for amplification were designed to amplify the region of *bla*_*CphA*_ containing the randomized codon of interest. The PCR primers also contained a 7 bp-barcode sequence that was unique for each library and each imipenem concentration used for selection. The primers were designed so that the PCR amplicons are approximately 150 bp in length. The resulting PCR products from each library and each imipenem selection were gel-purified and then pooled into a single tube with equal amounts. The pooled PCR products were ligated with adapters for sequencing and sequencing was performed using Illumina paired-end MiSeq sequencing (2 × 150 bp read length) by the Human Genome Sequencing Center at Baylor College of Medicine.

### Analysis of Illumina deep sequencing data

Illumina MiSeq sequencing returned FASTQ files containing the sequencing reads and quality information. The sequencing data was quality-checked using the Galaxy web server (https://usegalaxy.org/), which showed that read quality was high (scores over 30) for all nucleotide positions except those at the extreme end of the reads. A custom Perl script was developed to extract the mutant sequences for each library by using matches to the appropriate barcode sequence as well as the sequences 10 bp upstream and downstream of the randomized codon as described previously^18^.

### Creation of sequence logos

In order to determine and represent the predominant amino acid sequence types in the libraries after selection for antibiotic resistance, sequence logos were created using the algorithms described by Schneider *et al*.^35^. Briefly, total information content at each position *u* (*I*_*u*_) was calculated using the equations below:


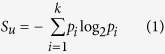



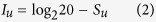


where *S* is the entropy, *p*_*i*_ stands for the fraction of times the *i*th type appears at a position, and *k* is the number of different amino acid residue types that appear at a position. Note that, for the *S* calculations, the value for fraction of times an amino acid residue type appears at a position (*p*_*i*_) was adjusted for the number of codons encoding the amino acid type. Self-information for each amino acid equals *I*_u_* *f*_*u,a*_, where *f*_*u,a*_ stands for occurrence frequency of the amino acid *a* at the position *u*. According to the calculation, in an experiment for a residue positon, if only one amino acid is observed at the positon, the information content for the experiment is 4.23 (log_2_20) bits and self-information for the amino acid is 4.23 bits as well. However, if each amino acid occurs at same frequency at the positon, the information content for the experiment is 0 bit. A Matlab script was used to create the sequence logo graphs.

### Protein expression and purification

The CphA-pET29a plasmid encoding wild-type or mutant CphA enzymes was used for protein expression in *E. coli* BL21 (DE3) cells. Wild-type CphA was expressed by growing the culture in LB medium containing 25 μg/ml kanamycin at 37 °C for 20 hr as described previously^4^. Expression of mutant CphA was achieved by inducing mid-log-phase cultures with 0.5 mM IPTG at 20 °C for 16 hr. Both wild-type and mutant CphA enzymes were purified to >95% homogeneity from the periplasmic fraction of the cells by using ion-exchange and gel-filtration chromatography as described by Bebrone *et al*.^19^. The purified enzyme was stored in 15 mM sodium cacodylate (pH 6.5) containing 0.1 mM ZnCl_2_ and quantified by measuring absorbance at 280 nm with a DU800 spectrophotometer (Beckman Coulter) and using the corresponding extinction coefficient (ε) for wild type or mutants, which were calculated using the ExPASy ProtParam tool (ε_280_ = 34, 380 M^−1^cm^−1^ for wild-type CphA).

### Determination of kinetic parameters

The determination of enzyme kinetic parameters for hydrolysis of imipenem by CphA or its mutants were performed at 30 °C in 15 mM sodium cacodylate (pH 6.5) buffer. Substrate hydrolysis was monitored with a DU800 spectrophotometer equipped with thermostatically controlled cells by following the change in absorbance at 300 nM (Δε_300nm_ = 9,000 M^−1^cm^−1^) resulting from opening of the β-lactam ring. Cuvettes with 0.1- or 1-cm path lengths were used, depending on the substrate concentrations. For wild type and most CphA mutants, *k*_*cat*_ and *K*_*m*_parameters were determined under initial-rate conditions by fitting the initial velocity (*v*_*o*_) at various substrate concentrations to the Michaelis-Menton equation (*v* = *V*_*max*_ [S]/(*K*_*m*_ + [S])) using GraphPad Prism5. When *V*_max_ could not be determined because *K*_*m*_ was too high, the catalytic efficiency (*k*_cat_/*K*_*m*_) was determined by analyzing the complete hydrolysis time courses at low imipenem concentration and fitting the data to equation *v* = *k*_*cat*_/*K*_*m*_ [E][S]^13^,^36^. Kinetic parameters were averaged from at least two independent determinations using 8 different substrate concentrations.

### Imipenem resistance determination

Imipenem resistance levels of *E. coli* XL1-Blue expressing CphA or its mutants were determined by measuring the minimum inhibitory concentration (MIC) of imipenem using the Etest method (bioMérieux) according to the manufacturer’s recommendations.

### Protein expression level determination

The effect of amino acid substitutions on steady-state expression of CphA in *E. coli* was determined according to Horton *et al*.^14^. Briefly, exponential phase cells (OD_600_ = 0.7–0.8) were collected by centrifugation and lysed in B-PER (Thermo Scientific) containing 0.1 mg/ml lysozyme and 0.02 mg/ml DNaseI. Equal volumes of cell lysates were subjected to SDS-PAGE and used for Western blotting hybridization. Because a StrepII tag was fused to the C-terminus of wild-type or mutant CphA, their expression was detected by probing with horseradish peroxidase (HRP)-conjugated mouse monoclonal anti-StrepII antibody (Novogen). In addition, the same membrane was also probed with antibody against DnaK (Enzo Life Sciences), which serves as a loading control. The hybridization signal was quantified by densitometry using ImageJ software (NIH).

## Additional Information

**How to cite this article**: Sun, Z. *et al*. Deep Sequencing of Random Mutant Libraries Reveals the Active Site of the Narrow Specificity CphA Metallo-β-Lactamase is Fragile to Mutations. *Sci. Rep.* **6**, 33195; doi: 10.1038/srep33195 (2016).

## Supplementary Material

Supplementary Information

Supplementary Table S1

## Figures and Tables

**Table 1:** Impact of amino acid substitutions on CphA function in imipenem hydrolysis and resistance.

Category	Residueposition	Amino acid substitution	MIC_IMP_ (μg/ml)	Imipenem hydrolysis kinetics^a^		
				*k*_*cat*_ (s^−1^)	*K*_*m*_ (μM)	*k*_*cat*_/*K*_*m*_ (s^−1^ μM^−1^)
		**WT**	2	2144	310	6.91
**Group 1**	**Gly84**	**Ala**	0.5	510	119	4.35
		**Asp**	0.5	28	209	0.14
	**Thr197**	**Leu**	0.38	40	308	0.13
		**Ser**	0.75	1512	371	4.10
	**Asp199**	**Glu**	0.75	365	188	1.95
	**Lys224**	**Arg**	0.25	>440	>800	0.87
	**Gly232**	**Ala**	0.5	>274	>800	0.77
	**Asn233**	**Ser**	1	1802	636	2.86
		**Gln**	0.5	371	633	0.59
	**Gly262**	**Ala**	0.38	241	547	0.44
	**His263**	**Ala**	0.38	>5	>800	0.012
**Group 2**	**Glu69**	**Asp**	0.38	64	388	0.17
		**Ser**	1	641	525	1.23
	**Tyr117**	**Phe**	1	177	216	0.83
		**Val**	0.5	>145	>800	0.35
		**Trp**	1	365	245	1.49
	**Arg121**	**Ala**	0.25	14	117	0.12
		**Lys**	0.75	>750	>800	3.38
	**Asn220**	**Gly**	0.75	562	84	6.70
		**Leu**	0.38	1.63	221	0.007
		**Gln**	0.5	796	164	4.90
**Group 3**	**Ala195**	**Gly**	1.5	ND	ND	ND
	**Pro198**	**Glu**	2	887	182	4.95
	**Ser235**	**Gly**	2	ND	ND	ND

^a^Data are mean results of at least two independent experiments and standard deviations are within 20% of the means; ND, not determined.

## References

[b1] Palzkill T. Metallo-β-lactamase structure and function. Ann. N. Y. Acad. Sci. 1277, 91–104 (2013). 23163348 10.1111/j.1749-6632.2012.06796.x PMC3970115

[b2] Bebrone C. Metallo-β-lactamases (classification, activity, genetic organization, structure, zinc coordination) and their superfamily. Biochem. Pharmacol. 74, 1686–1701 (2007). 17597585 10.1016/j.bcp.2007.05.021

[b3] Massidda O., Rossolini G. M. & Satta G. The *Aeromonas hydrophila* cphA gene: molecular heterogeneity among class B metallo-β-lactamases. J. Bacteriol. 173, 4611–4617 (1991). 1856163 10.1128/jb.173.15.4611-4617.1991 PMC208136

[b4] Hernandez Valladares M. . Zn(II) dependence of the *Aeromonas hydrophila* AE036 metallo-β-lactamase activity and stability. Biochemistry 36, 11534–11541 (1997). 9298974 10.1021/bi971056h

[b5] Felici A. . An overview of the kinetic parameters of class B β-lactamases. Biochem. J. 291 (Pt 1), 151–155 (1993). 8471035 10.1042/bj2910151 PMC1132494

[b6] Segatore B., Massidda O., Satta G., Setacci D. & Amicosante G. High specificity of *cphA*-encoded metallo-β-lactamase from *Aeromonas hydrophila* AE036 for carbapenems and its contribution to β-lactam resistance. Antimicrob. Agents Chemother. 37, 1324–1328 (1993). 8328781 10.1128/aac.37.6.1324 PMC187960

[b7] Garau G. . A metallo-β-lactamase enzyme in action: crystal structures of the monozinc carbapenemase CphA and its complex with biapenem. J.Mol. Biol. 345, 785–795 (2005). 15588826 10.1016/j.jmb.2004.10.070

[b8] Xu D., Xie D. & Guo H. Catalytic mechanism of class B2 metallo-β-lactamase. J. Biol. Chem. 281, 8740–8747 (2006). 16423823 10.1074/jbc.M512517200

[b9] Simona F. . Common mechanistic features among metallo-β-lactamases: a computational study of *Aeromonas hydrophila* CphA enzyme. J. Biol. Chem. 284, 28164–28171 (2009). 19671702 10.1074/jbc.M109.049502 PMC2788867

[b10] Wu S., Xu D. & Guo H. QM/MM studies of monozinc β-lactamase CphA suggest that the crystal structure of an enzyme-intermediate complex represents a minor pathway. J Am. Chem Soc. 132, 17986–17988 (2010). 21138257 10.1021/ja104241g PMC3009838

[b11] Bebrone C. . Dramatic broadening of the substrate profile of the *Aeromonas hydrophila* CphA metallo-β-lactamase by site-directed mutagenesis. J. Biol. Chem. 280, 28195–28202 (2005). 15863831 10.1074/jbc.M414052200

[b12] Bebrone C. . Mutational analysis of the zinc- and substrate-binding sites in the CphA metallo-β-lactamase from *Aeromonas hydrophila*. Biochem. J. 414, 151–159 (2008). 18498253 10.1042/BJ20080375

[b13] Brown N. G., Horton L. B., Huang W., Vongpunsawad S. & Palzkill T. Analysis of the functional contributions of Asn233 in metallo-β-lactamase IMP-1. Antimicrob. Agents Chemother. 55, 5696–5702 (2011). 21896903 10.1128/AAC.00340-11 PMC3232802

[b14] Horton L. B. . Mutagenesis of zinc ligand residue Cys221 reveals plasticity in the IMP-1 metallo-β-lactamase active site. Antimicrob. Agents Chemother. 56, 5667–5677 (2012). 22908171 10.1128/AAC.01276-12 PMC3486559

[b15] Vanhove M. . Role of Cys221 and Asn116 in the zinc-binding sites of the *Aeromonas hydrophila* metallo-β-lactamase. Cell. Mol. Life Sci. 60, 2501–2509 (2003). 14625692 10.1007/s00018-003-3092-x PMC11138725

[b16] Materon I. C., Beharry Z., Huang W., Perez C. & Palzkill T. Analysis of the context dependent sequence requirements of active site residues in the metallo-β-lactamase IMP-1. J. Mol. Biol. 344, 653–663 (2004). 15533435 10.1016/j.jmb.2004.09.074

[b17] Fowler D. M. & Fields S. Deep mutational scanning: a new style of protein science. Nat. Methods 11, 801–807 (2014). 25075907 10.1038/nmeth.3027 PMC4410700

[b18] Deng Z. . Deep sequencing of systematic combinatorial libraries reveals β-lactamase sequence constraints at high resolution. J. Mol. Biol. 424, 150–167 (2012). 23017428 10.1016/j.jmb.2012.09.014 PMC3524589

[b19] Bebrone C. . The structure of the dizinc subclass B2 metallo-β-lactamase CphA reveals that the second inhibitory zinc ion binds in the histidine site. Antimicrob. Agents Chemother. 53, 4464–4471 (2009). 19651913 10.1128/AAC.00288-09 PMC2764157

[b20] Adkar B. V. . Protein model discrimination using mutational sensitivity derived from deep sequencing. Structure 20, 371–381 (2012). 22325784 10.1016/j.str.2011.11.021

[b21] Fowler D. M. . High-resolution mapping of protein sequence-function relationships. Nat. Methods 7, 741–746 (2010). 20711194 10.1038/nmeth.1492 PMC2938879

[b22] Hinkley T. . A systems analysis of mutational effects in HIV-1 protease and reverse transcriptase. Nat. Genet. 43, 487–489 (2011). 21441930 10.1038/ng.795

[b23] Jiang L., Mishra P., Hietpas R. T., Zeldovich K. B. & Bolon D. N. Latent effects of Hsp90 mutants revealed at reduced expression levels. Plos Genet. 9, e1003600 (2013). 23825969 10.1371/journal.pgen.1003600 PMC3694843

[b24] Stiffler M. A., Hekstra D. R. & Ranganathan R. Evolvability as a function of purifying selection in TEM-1 β-lactamase. Cell 160, 882–892 (2015). 25723163 10.1016/j.cell.2015.01.035

[b25] Haruta S., Yamamoto E. T., Eriguchi Y. & Sawai T. Characterization of the active-site residues asparagine 167 and lysine 161 of the IMP-1 metallo β-lactamase. FEMS Microbiol. Lett. 197, 85–89 (2001). 11287151 10.1111/j.1574-6968.2001.tb10587.x

[b26] Yang Y., Keeney D., Tang X., Canfield N. & Rasmussen B. A. Kinetic properties and metal content of the metallo-β-lactamase CcrA harboring selective amino acid substitutions. J. Biol. Chem. 274, 15706–15711 (1999). 10336469 10.1074/jbc.274.22.15706

[b27] Zhang Z., Yu Y., Musser J. M. & Palzkill T. Amino acid sequence determinants of extended spectrum cephalosporin hydrolysis by the class C P99 β-lactamase. J. Biol. Chem. 276, 46568–46574 (2001). 11591698 10.1074/jbc.M102757200

[b28] Fisher J. F. & Mobashery S. Three decades of the class A β-lactamase acyl-enzyme. Curr. Protein Pept. Sci. 10, 401–407 (2009). 19538154 10.2174/138920309789351967 PMC6902449

[b29] Miller B. G. & Wolfenden R. Catalytic proficiency: the unusual case of OMP decarboxylase. Annu. Rev. Biochem. 71, 847–885 (2002). 12045113 10.1146/annurev.biochem.71.110601.135446

[b30] Yuan J., Cardenas A. M., Gilbert H. F. & Palzkill T. Determination of the amino acid sequence requirements for catalysis by the highly proficient orotidine monophosphate decarboxylase. Protein Sci. 20, 1891–1906 (2011). 21898650 10.1002/pro.728 PMC3267953

[b31] Petrosino J., Rudgers G., Gilbert H. & Palzkill T. Contributions of aspartate 49 and phenylalanine 142 residues of a tight binding inhibitory protein of β-lactamases. J. Biol. Chem. 274, 2394–2400 (1999). 9891008 10.1074/jbc.274.4.2394

[b32] Zheng L., Baumann U. & Reymond J. L. An efficient one-step site-directed and site-saturation mutagenesis protocol. Nucleic Acids Res. 32, e115 (2004). 15304544 10.1093/nar/gnh110 PMC514394

[b33] Huang W., Petrosino J., Hirsch M., Shenkin P. S. & Palzkill T. Amino acid sequence determinants of β-lactamase structure and activity. J. Mol. Biol. 258, 688–703 (1996). 8637002 10.1006/jmbi.1996.0279

[b34] Sullivan B., Walton A. Z. & Stewart J. D. Library construction and evaluation for site saturation mutagenesis. Enzyme Microb. Technol. 53, 70–77 (2013). 23683706 10.1016/j.enzmictec.2013.02.012

[b35] Schneider T. D. & Stephens R. M. Sequence logos: a new way to display consensus sequences. Nucleic Acids Res. 18, 6097–6100 (1990). 2172928 10.1093/nar/18.20.6097 PMC332411

[b36] Mehta S. C., Rice K. & Palzkill T. Natural variants of the KPC-2 carbapenemase have evolved increased catalytic efficiency for ceftazidime hydrolysis at the cost of enzyme stability. Plos Pathog. 11, e1004949 (2015). 26030609 10.1371/journal.ppat.1004949 PMC4452179

