# Supplementary material for: Deep Sequencing of Random Mutant Libraries Reveals the Active Site of the Narrow Specificity CphA Metallo-β-Lactamase is Fragile to Mutations
Source: Sci Rep. 2016 Sep 12;6:33195. doi: 10.1038/srep33195 (PMC5018959; doi:10.1038/srep33195)
Supplement: Supplementary Information [file srep33195-s1.pdf]

Supplementary information:

Deep Sequencing of Random Mutant Libraries Reveals the Active Site of the Narrow Specificity CphA Metallo-β-Lactamase is Fragile to Mutations

Zhizeng Sun[1], Shrenik C. Mehta[1], Carolyn Adamski[2], Richard A. Gibbs[3], Timothy Palzkill[1*]

From the [1]Department of Pharmacology, [2]Department of Biochemistry and Molecular Biology, [3]Human Genome Sequencing Center, Baylor College of Medicine, One Baylor Plaza, Houston, TX 77030, USA

[*]To whom correspondence should be addressed: One Baylor Plaza, Houston, TX 77030. Tel.: 713-798-5609; Fax: 713-798-7375; E-mail: timothyp@bcm.edu.

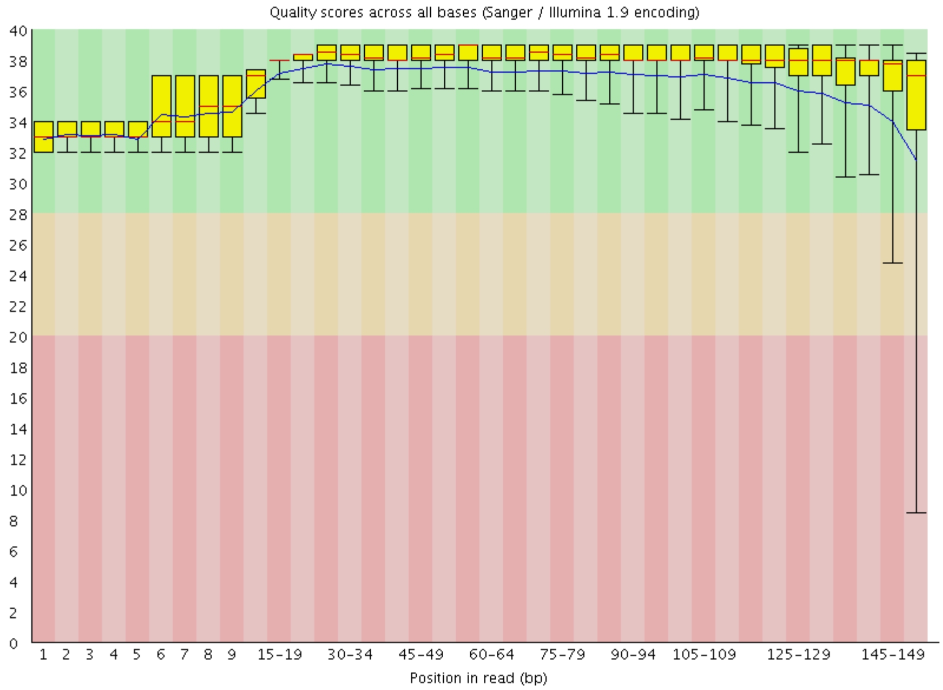

FIGURE S1. Quality scores across all sequenced bases created by the Galaxyweb server. The file containing sequencing data was uploaded onto https://usegalaxy.org/ and the FASTQ groomer was run to convert the FASTQ file to standard format. FastQC Read Quality reports were produced which includes per base sequence quality, per sequence quality scores, per base sequence content and sequence length distribution. Here, only per base sequence quality is shown as a BoxWhisker type plot to indicate the average quality score at each position across all reads. In the graph, the x-axis shows the position in the sequencing read and the y-axis shows the quality scores of the corresponding positions. The higher the score the better the base call. The background of the graph divides the y axis into calls of very good quality (green), calls of reasonable quality (orange), and calls of poor quality (red). The yellow box represents the inter-quartile range (25-75%) and the central red line represents the median value of the quality score. The upper and lower whiskers represent the 10% and 90% points of the quality scores, respectively. The blue line represents the mean quality.

TABLE S1. Occurrence of each amino acid type at each randomized position in naïve and 0.1, 0.2, 0.4 and 0.8 μg/ml imipenem-selected libraries. Wild-type amino acid types are labelled red in the corresponding position. See Table 1.xls Excel dataset file.

TABLE S2. Summary of tolerance of active site residues to amino acid substitutions.

Enzyme	Amino acid residue positions tested[a]	Reference
CphA β-lactamase	V67, Q68, E69, G84, W87, N116, Y117, H118, D120, R121, P194, A195, H196, T197, P198, D199, N220, C221, K224, E225, G232, N233, S235, F236, G262, H263	This work
IMP-1 β-lactamase	V61, N62, G63, W64, G65, V67, P68, K69, D84, F87, L95, H116, H118, D120, G123, L145, P194, H196, T197, T199, C221, K224, P225, G228, G232, N233, D236, S262, H263	Materon et al., 2004[1]
TEM-1 β-lactamase	M68, M69, S70, T71, F72, K73, V74, E104, Y105, S106, M129, S130, D131, N132, T133, P167, E168, L169, N170, K215, V216, A217, G218, P219, K234, S235, G236, A237, G238, E240, R241	Stiffler et al., 2015[2]
P99 β-lactamase	S64, K67, L119, Q120, Y150, N152, R204, D217, A220, Y221, E272, S289, L293, K315, T316, G317, S318, T319, S343, N346, R349	Zhang et al., 2001[3]
ODCase (*E. coli*)	V18, A20, D22, K44, K47, E48, D71, K73, D76, T80, H83, G103, V127, L130, T131, V167, C168, V187, P189, G190, Q201, V219, R222, P223	Yuan et al., 2011[4]

[a] Residues colored in red do not tolerate amino acid substitutions in selections for wild-type levels of function.

REFERENCES

1 Materon, I. C., Beharry, Z., Huang, W., Perez, C. & Palzkill, T. Analysis of the context dependent sequence requirements of active site residues in the metallo-β-lactamase IMP-1. *J. Mol. Biol.* **344**, 653-663 (2004).

2 Stiffler, M. A., Hekstra, D. R. & Ranganathan, R. Evolvability as a function of purifying selection in TEM-1 β-lactamase. *Cell* **160**, 882-892 (2015).

3 Zhang, Z., Yu, Y., Musser, J. M. & Palzkill, T. Amino acid sequence determinants of extended spectrum cephalosporin hydrolysis by the class C P99 β-lactamase. *J. Biol. Chem.* **276**, 46568-46574 (2001).

4 Yuan, J., Cardenas, A. M., Gilbert, H. F. & Palzkill, T. Determination of the amino acid sequence requirements for catalysis by the highly proficient orotidine monophosphate decarboxylase. *Protein Sci.* **20**, 1891-1906 (2011).
